# Adaptive Mitochondrial Specialization in Lung Adenocarcinoma: Redox Homeostasis, Hypoxia Adaptation, and Therapeutic Resistance

**DOI:** 10.3390/antiox15101364

**Published:** 2026-10-21

**Authors:** Manuel Castillejos-López, Ulises Barrera-Elizalde, Edgar Flores-Soto, Luz A. Colín-Godínez, Luz María Torres-Espíndola, Bianca S. Romero-Martinez, Joel Guzmán-Castillo, Georgina González-Avila, Erika Rubí Luis-García, Fausto Rojas-Duran, Jesús Antonio Lara-Reyes, Víctor Ruiz, Arnoldo Aquino-Gálvez

**Affiliations:** 1 Laboratorio de Investigación en Epidemiologia e Infectología, Instituto Nacional de Enfermedades Respiratorias Ismael Cosío Villegas (INER), Mexico City 14080, Mexico; manuel.castillejos@iner.gob.mx; 2 Laboratorio de Biología Molecular, Departamento de Investigación en Fibrosis Pulmonar, Instituto Nacional de Enfermedades Respiratorias Ismael Cosío Villegas (INER), Mexico City 14080, Mexico; ulises.barrera@comunidad.unam.mx (U.B.-E.); mcoling2300@alumno.ipn.unam.mx (L.A.C.-G.); jguzmanc2301@alumno.ipn.mx (J.G.-C.); 3 Posgrado en Ciencias Biológicas, Facultad de Medicina, Universidad Nacional Autónoma de México (UNAM), Mexico City 04510, Mexico; 4 Departamento de Farmacología, Facultad de Medicina, Universidad Nacional Autónoma de México (UNAM), Mexico City 04510, Mexico; eflores@facmed.unam.mx (E.F.-S.); biancasromero_@hotmail.com (B.S.R.-M.); 5 Laboratorio de Farmacología, Instituto Nacional de Pediatría (INP), Mexico City 04530, Mexico; ltorrese@pediatria.gob.mx; 6 Departamento de Investigación en Enfermedades Crónico Degenerativas, Instituto Nacional de Enfermedades Respiratorias Ismael Cosío Villegas (INER), Mexico City 14080, Mexico; georgina.gonzalez@iner.gob.mx; 7 Laboratorio de Biología Celular, Departamento de Investigación en Fibrosis Pulmonar, Instituto Nacional de Enfermedades Respiratorias Ismael Cosío Villegas (INER), Mexico City 14080, Mexico; erika.luis@iner.gob.mx; 8 Instituto de Investigaciones del Cerebro, Universidad Veracruzana, Xalapa 91190, Mexico; frojas@uv.mx (F.R.-D.); jlara@uv.mx (J.A.L.-R.); 9 Departamento de Posgrado, Escuela Superior de Medicina, Instituto Politécnico Nacional, Mexico City 11340, Mexico; 10 Departamento de Bioquímica, Facultad de Medicina, Universidad Nacional Autónoma de México (UNAM), Mexico City 04510, Mexico

**Keywords:** lung adenocarcinoma, hypoxia, redox homeostasis, reactive oxygen species (ROS), mitochondrial specialization, mitochondrial proteostasis, cancer cachexia, metabolic reprogramming, immunotherapy, therapeutic resistance

## Abstract

Lung adenocarcinoma (LUAD) exhibits metabolic flexibility that transcends the classical interpretation of the Warburg effect. This review proposes the concept of “adaptive mitochondrial specialization (AMS)”, an integrative framework in which mitochondria undergo coordinated, context-dependent remodeling of bioenergetic metabolism, redox homeostasis, architecture and dynamics, proteostasis, mitochondrial genome maintenance and translation, and inter-organelle communication in response to hypoxia and other tumor-associated stressors. We examine how hypoxia can promote this adaptive state through HIF-dependent metabolic reprogramming together with intrinsic mitochondrial stress responses, including respiratory-chain remodeling, proteostatic regulation, and context-dependent control of reactive oxygen species (ROS). We further discuss mitochondrial genome and translational maintenance, fusion/fission dynamics, apoptotic susceptibility, and mitochondria-associated membrane (MAM) signaling as mechanisms through which mitochondrial function can be adjusted to environmental and therapeutic stress. Beyond tumor cells, we examine how mitochondrial quality-control alterations accompany systemic metabolic remodeling during cancer cachexia and how impaired mitophagy and intercellular mitochondrial transfer can influence antitumor T-cell function. These tumor–host manifestations are considered consequences or interacting mechanisms of cancer-associated mitochondrial remodeling rather than defining criteria of AMS. Finally, we discuss how context-dependent mitochondrial and redox dependencies may expose therapeutic vulnerabilities and provide a rationale for bioenergetic stratification in precision oncology.

## 1. Introduction

Lung adenocarcinoma (LUAD) remains one of the leading causes of cancer mortality worldwide, characterized by remarkable genetic heterogeneity and exceptional adaptive capacity in the face of pressures from the tumor microenvironment. Traditionally, cancer metabolic reprogramming has been interpreted through the Warburg effect, suggesting preferential aerobic glycolysis and, consequently, reduced tumor-cell reliance on mitochondrial oxidation. However, emerging evidence challenges this paradigm, as the current model does not fully explain the metabolic changes and redox adaptation observed in LUAD.

Under microenvironmental conditions such as hypoxia, nutrient limitation, and oxidative stress, tumor-cell mitochondria can undergo extensive functional remodeling rather than becoming globally inactive. This remodeling can affect bioenergetics, redox signaling, biosynthesis, apoptosis, mitochondrial dynamics, and cellular plasticity. For example, MTP18 (also designated as MTFP1, mitochondrial fission process 1) induces excessive mitochondrial fission that activates the oncogenic phosphoinositide 3-kinase/protein kinase B (PI3K/AKT) pathway, promoting proliferation, migration, and invasion in LUAD [1].

Hypoxia-inducible factors (HIFs) coordinate transcriptional responses to reduced oxygen availability, while complementary mitochondrial proteostatic mechanisms contribute to adaptation under metabolic stress. In NSCLC, TNF receptor-associated protein 1 (TRAP1) supports mitochondrial function, ATP production and tumor cell survival [2]. In complementary mechanistic models, the mitochondrial protease YME1L mediates stress-responsive proteolytic remodeling of the mitochondrial proteome in response to hypoxia or nutrient limitation [3]. Similarly, mitochondrial association of hexokinase II (HK2) can couple metabolic activity to apoptotic susceptibility [4]. Mitochondrial gene expression and genome maintenance provide an additional layer of functional preservation: mitochondrial RNA polymerase (POLRMT) is required for mitochondrial gene expression and is overexpressed in LUAD [5], whereas deoxyguanosine kinase (DGUOK) supports mtDNA homeostasis, respiratory Complex I biogenesis, OXPHOS, and cancer stem-cell maintenance in lung adenocarcinoma [6].

In this review, we integrate these adaptive mechanisms within the proposed framework of adaptive mitochondrial specialization (AMS). In this context, rather than assuming global mitochondrial suppression under hypoxia or oncogenic stress, AMS describes coordinated and context-dependent remodeling of mitochondrial metabolism, architecture, proteostasis, redox control, and inter-organelle communication that preserves functions compatible with tumor-cell adaptation.

In LUAD, the tumor’s genomic profile may shape this specialization, enabling context-dependent metabolic responses to nutrient deprivation. In tumors with an inactivation of serine/threonine kinase 11 (STK11/LKB1), increased expression of the mitochondrial enzyme carbamoyl-phosphate synthase 1 (CPS1) supports metabolic programs associated with nucleotide biosynthesis and tumor-cell proliferation [7,8]. Furthermore, under hypoxic conditions, tumors with a Kirsten rat sarcoma viral oncogene homolog (*KRAS*) mutation suppress phospholipase C gamma 1 (PLCγ1), limiting calcium (Ca2+) influx into the mitochondrial matrix. This adjustment reduces mitochondrial reactive oxygen species (ROS) and lipid peroxidation, antagonizes apoptosis, and shifts tumor bioenergetics toward glycolysis [9].

Finally, mitochondrial remodeling can influence therapeutic response through several distinct mechanisms. In epidermal growth factor receptor (*EGFR*)-mutated LUAD, mitochondrial apoptotic signaling involving Bit1 has been implicated in the response to EGFR tyrosine kinase inhibitors; however, Bit1-mediated anoikis and EGFR-TKI resistance should be considered mechanistically distinct contexts [10]. In addition, mitophagy-related genes, including translocase of outer mitochondrial membrane 40 (TOMM40) and voltage-dependent anion channel 1 (VDAC1), have been associated with LUAD prognosis and tumor-microenvironment features, supporting the potential clinical relevance of mitophagy-related programs without establishing a direct causal role in treatment resistance [11]. In this context, iron handling, iron–sulfur cluster biogenesis, and oxidative-stress control represent interconnected metabolic and redox processes that may expose therapeutic vulnerabilities in treatment-resistant LUAD [12].

Overall, these observations support the concept that coordinated mitochondrial remodeling contributes to metabolic plasticity, redox balance, tumor progression, and therapeutic resistance. In this review, we examine hypoxia-mediated mitochondrial restructuring that contributes to LUAD pathophysiology, and propose AMS as an integrative operational framework for coordinated adaptations in bioenergetics, redox signaling, proteome remodeling, inter-organelle communication, and therapeutic adaptation.

### 1.1. Literature Search Strategy

A literature search was conducted in PubMed/MEDLINE for studies published between October 2006 and October 2026. Search terms included lung adenocarcinoma in combination with terms related to mitochondrial metabolism and proteome remodeling, hypoxia, metabolic plasticity, OXPHOS, ROS and redox regulation, mitochondrial dynamics and quality control, mtDNA and mitochondrial translation, inter-organelle communication, and therapy resistance.

Priority was given to original mechanistic studies in LUAD. When LUAD-specific evidence was limited, relevant studies in NSCLC or other tumor models were included to support specific mitochondrial mechanisms. At the time, review articles were used for broader context and the identification of primary literature. Studies were selected according to their relevance to the main components of the proposed adaptive mitochondrial specialization (AMS) framework. The search was last updated in October 2026 to incorporate recent evidence.

### 1.2. Operational Definition of AMS

Operationally, adaptive mitochondrial specialization (AMS) is proposed here as an adaptive mitochondrial response induced by hypoxia, nutrient restriction, or other stressors within the tumor microenvironment, wherein the mitochondrion undergoes coordinated remodeling of its metabolism, architecture, dynamics, proteostasis, redox homeostasis, and inter-organelle communication while preserving bioenergetic, biosynthetic, and survival functions compatible with tumor cell adaptation. This operational definition integrates established mitochondrial roles in bioenergetics, biosynthesis, redox homeostasis, and metabolic adaptation [13] with stress-responsive mitochondrial remodeling mechanisms discussed throughout this review. Unlike a simplified interpretation of the Warburg effect—which emphasizes high glucose utilization and lactate production even in the presence of functional mitochondria—AMS does not entail global mitochondrial inactivation, but rather functional, selective reprogramming aligned with environmental demands and the tumor’s genetic context [14].

AMS is operationalized through the integration of four functional domains: (1) adaptive bioenergetic reprogramming, characterized by the preservation of residual or functionally sufficient mitochondrial capacity—including OXPHOS—in dynamic coexistence with glycolysis and other nutrient utilization pathways, such as fatty acid oxidation and glutaminolysis [15,16]; (2) control of redox homeostasis through the coordinated regulation of ROS production and detoxification, the availability of reducing equivalents, and the control of lipid peroxidation, maintaining a balance between adaptive redox signaling and the prevention of oxidative damage [17,18,19]; (3) remodeling of mitochondrial architecture, dynamics, and proteostasis, including proteome modifications, maintenance of mtDNA integrity and mitoribosomal function, regulation of fusion and fission processes, and mitochondrial quality control mechanisms [3,6,20]; and (4) context-dependent metabolic and inter-organelle integration, comprising the remodeling of endoplasmic reticulum–mitochondria communication—particularly at mitochondria-associated membranes (MAMs)—alongside the regulation of Ca^2+^ signaling, lipid metabolism, and other processes coordinated at ER–mitochondrial interfaces [21], together with context-dependent nutrient utilization shaped by the tumor’s genetic background and environmental stress [22].

The resulting specialization is shaped by the interaction between the tumor’s genetic background and environmental stress. In LUAD, distinct oncogenic and tumor-suppressor alterations impose different metabolic and redox requirements and may therefore favor the engagement of different components of the proposed AMS program. LKB1/STK11 inactivation is associated with increased expression of the mitochondrial enzyme CPS1 and CPS1-dependent metabolic programs that support tumor-cell growth and nucleotide metabolism [8]. In KRAS-mutant LUAD, hypoxic suppression of PLCγ1 reduces mitochondrial Ca^2+^ uptake and mitochondrial ROS production, limits lipid peroxidation, and shifts tumor bioenergetics toward glycolysis, illustrating how genotype and environmental stress can converge on mitochondrial adaptation [9]. Moreover, co-occurring LKB1 loss and KEAP1/NRF2 activation promote energetic and redox adaptation by increasing dependence on glutamine metabolism, while KEAP1 loss creates glutaminolytic dependency in KRAS-driven LUAD [22,23]. These examples illustrate that AMS is not a uniform molecular phenotype defined by a fixed set of proteins or pathways, but rather a context-dependent mitochondrial specialization in which distinct genetic backgrounds can engage different adaptive mechanisms while converging on the preservation of bioenergetic, redox, and survival functions.

Overall, AMS is not defined by the isolated presence of elevated glycolysis, OXPHOS, redox signaling, proteostatic remodeling, MAM alterations, or dependence on a specific substrate. Instead, it is defined by their coordinated integration into a stress-induced adaptive mitochondrial program associated with preserved mitochondrial function, containment of oxidative damage, suppression of stress-induced cell-death programs, and maintenance of tumor cell fitness. Therefore, supporting AMS requires evidence of a coordinated adaptive response and cannot be inferred from an isolated alteration in a metabolic, redox, structural, or proteostatic marker.

In this sense, AMS is intended to complement, rather than replace, established concepts of metabolic plasticity, mitochondrial reprogramming, and hybrid glycolytic/OXPHOS states. Metabolic plasticity broadly describes tumor cells’ ability to shift among metabolic pathways in response to intrinsic or environmental demands, whereas the hybrid glycolytic/OXPHOS phenotype refers more specifically to the concurrent use of glycolysis and oxidative metabolism [15,16]. Mitochondrial reprogramming encompasses adaptive changes in mitochondrial metabolism and function that support tumor bioenergetic and biosynthetic demands [15]. AMS builds on these concepts by proposing that under defined environmental or therapeutic stress, mitochondrial adaptation becomes functionally integrated across the bioenergetic, redox, structural/proteostatic, and inter-organelle domains. Accordingly, the AMS framework proposes that this state should be supported by coordinated, context-dependent changes across multiple mitochondrial domains, rather than by a single metabolic phenotype or molecular marker, and that the disruption of critical nodes within this integrated network may expose stress-specific vulnerabilities not predicted by measurements of glycolysis or OXPHOS alone.

Conversely, isolated metabolic switching without coordinated mitochondrial remodeling, or mitochondrial alterations that primarily reflect irreversible organelle failure rather than an adaptive response, would not by themselves fulfill the operational criteria for AMS.

## 2. Hypoxia as the Driver of Adaptive Mitochondrial Specialization

**Figure 1:**
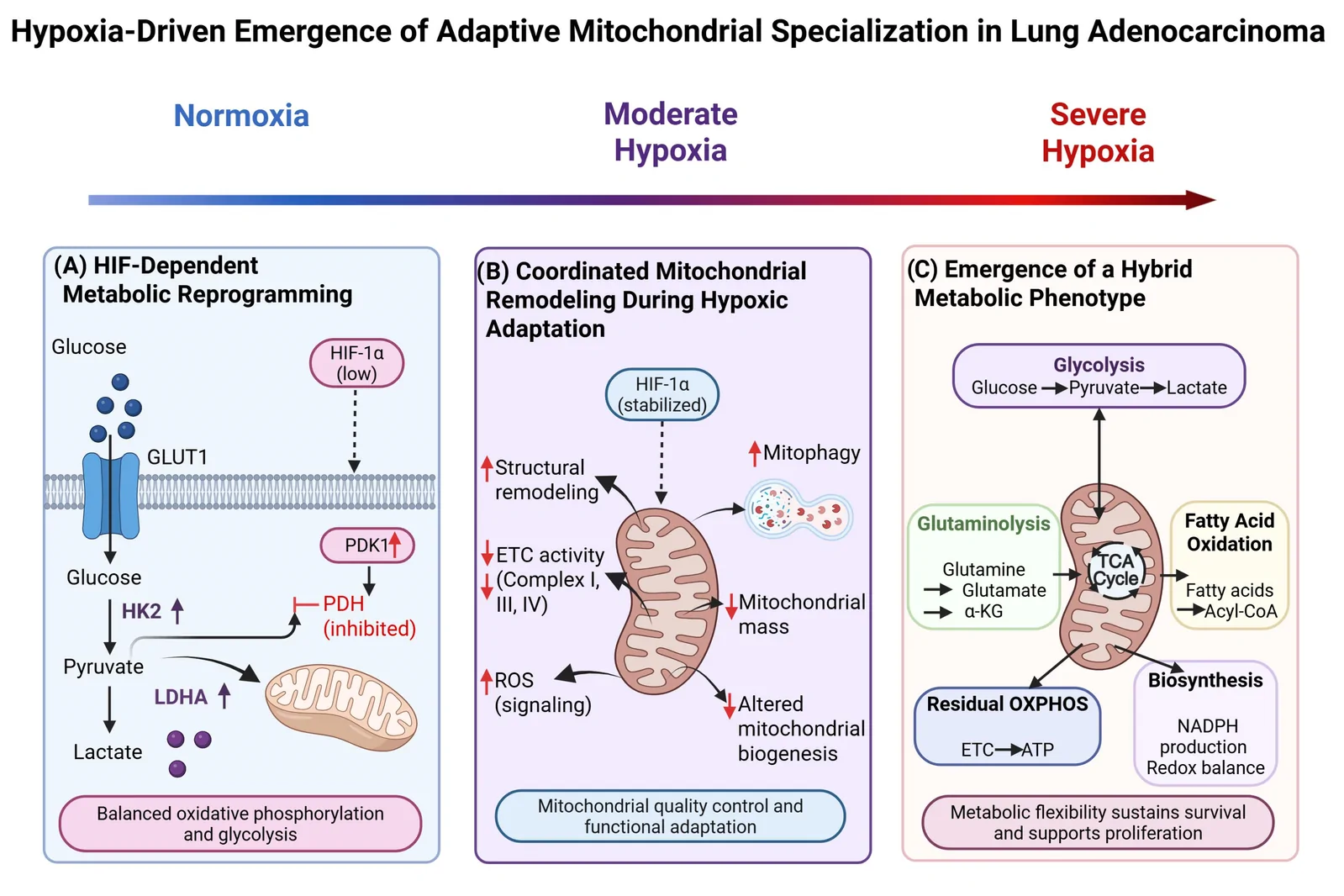
Hypoxia-driven emergence of AMS in lung adenocarcinoma. The figure provides a conceptual representation of the progressive metabolic and mitochondrial adaptations associated with increasing hypoxic stress. It should not be interpreted as three discrete metabolic states restricted to fixed O_2_ concentrations. (**A**) HIF-dependent metabolic reprogramming illustrates a progressive shift from a relatively balanced contribution of glycolysis and oxidative phosphorylation toward increased glucose uptake and glycolytic flux, along with restricted pyruvate entry into mitochondrial oxidation as oxygen availability decreases. (**B**) As hypoxic stress increases, HIF-dependent signaling and intrinsic mitochondrial stress responses promote coordinated remodeling of respiratory-chain activity, ROS signaling, mitochondrial structure, mitophagy, and mitochondrial mass. (**C**) Under more severe hypoxic stress, metabolic plasticity may support a hybrid phenotype in which enhanced glycolysis coexists with residual OXPHOS, glutaminolysis, fatty-acid oxidation, and biosynthetic and redox-supporting pathways. For this schematic, moderate hypoxia is operationally represented as approximately 0.5–3% O_2_, whereas severe hypoxia refers to <0.5% O_2_ [24,25]. These ranges are approximate operational categories derived from the tumor-hypoxia literature rather than universal intratumoral thresholds, because oxygen availability varies spatially and temporally within tumors and across experimental models. Created in BioRender. Aquino-gálvez, A. (2026) https://BioRender.com/hifgw3f.

The LUAD microenvironment is characterized by hypoxia, which represents an important selective pressure during tumor progression. Instead of simply limiting tumor-cell growth, reduced oxygen availability can select for or promote metabolically flexible subpopulations that sustain critical processes such as proliferation, survival, and dissemination while coping with altered oxidative stress. Hypoxia is proposed as a central environmental driver of AMS, promoting coordinated transcriptional, redox, metabolic, and structural responses (Figure 1).

### 2.1. HIF-Dependent Metabolic Reprogramming

Under hypoxic stress, HIFs are activated and coordinate metabolic reprogramming that promotes glycolysis and nutrient acquisition. In LUAD, this response can be modulated by the SWI/SNF chromatin-remodeling complex, which regulates the accessibility of promoters and enhancers of key metabolic genes. For example, loss of the SWI/SNF subunit ARID1A increases chromatin accessibility to HIF1α/BRD4, thereby promoting a glycolytic phenotype [26].

A critical adaptation observed in LUAD and other lung cancer models is the stabilization of the glutamine transporter solute carrier family 1 member 5 (SLC1A5) through the reduced activity of the ubiquitin ligase NEDD4 (neural precursor cell expressed, developmentally downregulated 4) [27,28], thereby promoting glutamine uptake to support bioenergetics, glutathione-mediated antioxidant defense, and cell growth. Furthermore, in non-small cell lung cancer (NSCLC), activation of the circ_0017521/PFKFB3 axis has been shown to promote glycolysis and epithelial–mesenchymal transition (EMT) [29].

Furthermore, tumor progression is linked to the dysregulation of glycerophospholipid metabolism. The loss of glycerol-3-phosphate dehydrogenase-like protein 1 (GPD1L) interferes with PHD-dependent HIF-1α hydroxylation, preventing its proteasomal degradation and promoting resistance to hypoxia and apoptosis [30]. However, this adaptation reduces AKT phosphorylation, downregulating the nuclear factor erythroid 2-related factor 2/glutathione peroxidase 4 (NRF2/GPX4) axis. Although this response may enhance tolerance to hypoxia, reduced GPX4 expression may create vulnerability by increasing susceptibility to ferroptosis due to impaired lipid peroxide clearance, suggesting the GPX4/ferroptosis axis as a potential therapeutic vulnerability.

### 2.2. Coordinated Mitochondrial Remodeling During Hypoxic Adaptation

Hypoxic adaptation extends beyond glycolysis induction and involves multiple processes that remodel mitochondrial function. During AMS, HIF-dependent signaling and intrinsic mitochondrial stress responses can reshape mitochondrial metabolism, proteostasis, redox homeostasis, and bioenergetic efficiency under hypoxic conditions in ways that preserve functions compatible with cellular adaptation.

A key mechanism of hypoxia-induced mitochondrial remodeling involves NDUFA4L2, a HIF-1 target that attenuates mitochondrial oxygen consumption by inhibiting Complex I activity, thereby limiting ROS production under low-oxygen conditions [31]. NDUFA4L2 dysregulation has also been described in clear cell renal cell carcinoma and hepatocellular carcinoma [32,33], providing additional tumor-context evidence for this adaptive mechanism. Meanwhile, proteostatic mechanisms add another layer of mitochondrial adaptation. In NSCLC, the mitochondrial chaperone TRAP1 supports mitochondrial function, ATP production, and tumor-cell survival [2]. Likewise, in various cell models, mTORC1 inhibition during hypoxia or nutrient starvation initiates a lipid-signaling cascade that promotes YME1L-mediated proteolytic remodeling of the mitochondrial proteome, limiting mitochondrial biogenesis and supporting cell growth under metabolic stress [3].

Together, these mechanisms indicate that hypoxia does not simply suppress mitochondrial activity but promotes selective remodeling of mitochondrial function to preserve bioenergetic and redox homeostasis under oxygen-limited conditions. This adaptation requires not only the modulation of respiratory activity and mitochondrial proteostasis, but also coordinated control of ROS production, antioxidant buffering, and redox signaling. Within the proposed AMS framework, these processes contribute to its redox component and are discussed below.

### 2.3. Mitochondrial Redox Buffering and Redox Signaling

Mitochondrial redox homeostasis represents a central functional domain of the proposed adaptive mitochondrial specialization (AMS) framework, as tumor cells must maintain reactive oxygen species (ROS) within a range that supports adaptive signaling while limiting irreversible oxidative damage. In this context, redox adaptation does not mean completely suppressing mitochondrial ROS, but rather coordinating ROS production, detoxification, reducing capacity, and downstream signaling in response to metabolic and environmental demands.

Mitochondrial superoxide generated during electron transport is converted to hydrogen peroxide by superoxide dismutase 2 (SOD2/MnSOD). In LUAD, TNF-α-dependent inflammation increases SOD2 expression, whereas SOD2 inhibition reduces TNF-α-induced proliferation, supporting a context-dependent protumorigenic role for this mitochondrial antioxidant enzyme [34]. The mitochondrial thioredoxin–peroxiredoxin system, including TXN2, PRDX3, and TXNRD2, further regulates hydrogen peroxide. Experimental disruption of TXNRD2 alters mitochondrial redox balance, promotes PHD2 accumulation and HIF-1α degradation, decreases VEGF expression, and reduces tumor growth and vascularization [35]. Together, these mechanisms illustrate how mitochondrial ROS buffering can integrate with tumor-adaptive signaling rather than serving solely as a passive defense against oxidative damage.

Redox buffering is tightly coupled to the availability of reducing equivalents. Nicotinamide nucleotide transhydrogenase (NNT) links the mitochondrial proton gradient to NADPH production. In NSCLC, NNT supports mitochondrial metabolism and tumorigenic capacity by maintaining NADPH availability and protecting iron–sulfur (Fe–S) protein function. Notably, NNT loss causes mitochondrial dysfunction without a substantial increase in global oxidative stress; instead, it impairs of Fe–S-dependent protein activity through mechanisms linked to reduced NADPH availability and increased ROS-mediated oxidation of these cofactors [17]. Mitochondrial isocitrate dehydrogenase 2 (IDH2) provides an additional link between intermediary metabolism and reducing capacity; in lung cancer cells, IDH2 contributes to metabolic and redox regulation and promotes HIF-1α-dependent metabolic reprogramming and tumor growth [36]. Other NADPH-generating reactions may contribute in a context-dependent manner. For example, inhibition of mitochondrial malic enzyme 2 (ME2) decreases NADPH levels and increases ROS and oxidative stress [37]. However, these findings were obtained in acute myeloid leukemia and should therefore be regarded as mechanistic evidence rather than LUAD-specific evidence.

NADPH availability is functionally coupled to the glutathione redox pool. Glutathione reductase uses NADPH to regenerate reduced glutathione (GSH) from oxidized glutathione (GSSG), thereby sustaining the reducing capacity required for glutathione-dependent peroxide detoxification. Accordingly, the GSH/GSSG balance links NADPH-generating pathways to antioxidant buffering. Experimental analyses of tumor mitochondria support this coupling between mitochondrial NADPH supply, GSH/GSSG recycling, and glutathione peroxidase-dependent ROS control. However, these findings were obtained in hepatoma rather than LUAD [38].

Control of lipid peroxidation represents another layer of redox adaptation. The glutathione (GSH)–GPX4 system limits phospholipid peroxide accumulation and protects cells from ferroptotic death. In LUAD with acquired resistance to EGFR tyrosine kinase inhibitors (EGFR-TKIs), GPX4 is a therapeutic vulnerability, and inhibiting it promotes ferroptotic cell death in EGFR-TKI-resistant cells [19]. Importantly, mitochondrial involvement in ferroptosis is context-dependent: mitochondrial metabolism, including the tricarboxylic acid cycle and electron transport chain, contributes to lipid peroxide accumulation during cysteine-deprivation-induced ferroptosis but is not similarly required when GPX4 inhibition directly triggers ferroptosis [18]. Ferroptosis should therefore be regarded as a potential consequence of failed redox control rather than a defining feature of AMS.

At the transcriptional level, the KEAP1/NRF2 pathway can integrate redox stress with broader antioxidant and metabolic programs. In EGFR-mutant LUAD, AURKA-dependent modulation of the KEAP1/NRF2/HO-1 axis has been associated with changes in GPX4 expression and ferroptosis sensitivity [39]. However, KEAP1/NRF2 activation should be regarded as a context-dependent regulator rather than a defining criterion of AMS.

Taken together, the redox component of AMS is best understood as dynamic and compartmentalized redox control rather than maximal antioxidant activity or uniformly low ROS levels. The biological effects of ROS depend not only on their abundance, but also on their subcellular origin, duration, and local buffering capacity. Transient, sublethal ROS generated within defined mitochondrial or cellular compartments can function as adaptive signals, whereas sustained or insufficiently buffered ROS can exceed local antioxidant capacity and promote oxidative damage, lipid peroxidation, apoptosis, or ferroptosis [40,41]. Thus, apparently opposing observations—such as ROS suppression protecting tumor cells from oxidative death while localized or moderate ROS production supports protumorigenic signaling—are not mutually exclusive, but instead reflect the spatial, temporal, and concentration-dependent nature of redox regulation.

From an operational perspective, redox specialization within AMS should therefore be supported by convergent evidence of ROS regulation, antioxidant buffering, reducing capacity, lipid-peroxidation control, and redox signaling under a defined stress. Accordingly, no isolated alteration in SOD2, PRDX3/TXNRD2, NNT, IDH2, ME2, glutathione, GPX4, or NRF2 is sufficient to establish AMS; these responses must occur as part of a coordinated mitochondrial adaptive program.

### 2.4. Emergence of a Hybrid Metabolic Phenotype

Emerging evidence challenges the view that hypoxic tumor cells necessarily become completely dependent on glycolysis. Instead, cancer cells can exhibit metabolic plasticity in which enhanced glycolysis coexists with residual or functionally relevant mitochondrial oxidative metabolism, giving rise to a hybrid glycolytic/OXPHOS phenotype [15,16].

In this phenotype, glycolysis can provide ATP and biosynthetic intermediates. At the same time residual mitochondrial activity can continue to support redox homeostasis, nucleotide synthesis, amino acid metabolism, bioenergetics, and cell survival. Mitochondrial gene-expression and genome-maintenance pathways may contribute to preserving this functional capacity. POLRMT, an essential component of mitochondrial gene expression, is overexpressed in LUAD [5]. In contrast, DGUOK maintains mtDNA homeostasis, Complex I biogenesis, and OXPHOS and supports cancer stem-cell maintenance in lung adenocarcinoma [6]. PGC-1α, a regulator of mitochondrial biogenesis and oxidative metabolism, is elevated in p53-WT LUAD cells and supports their proliferation [42].

Adaptation extends beyond mitochondrial metabolism to include hypoxia-responsive mechanisms that support glycolysis and adaptation to extracellular acidosis. Increased HK2 can support glycolytic metabolism [43], whereas carbonic anhydrase 9 (CA9) contributes to pH regulation and survival under hypoxic and acidic conditions [44].

Meanwhile, hypoxia also modulates mitochondrial gene regulation. Under hypoxia, activation of JAK/STAT3 signaling promotes mitochondrial translocation of NF-κB RelA, where RelA binds mitochondrial DNA (mtDNA) G-quadruplexes and promotes their folding. This process increases mtDNA instability, inhibits mitochondrial transcription, and promotes a metabolic shift toward glycolysis [45]. Together, these mechanisms illustrate how hypoxic adaptation can increase glycolytic capacity while retaining context-dependent mitochondrial functions, thereby supporting the metabolic plasticity incorporated into the proposed AMS framework.

### 2.5. Hypoxia-Mediated Remodeling of the Tumor Microenvironment

Hypoxia extends beyond tumor cells and contributes to remodeling of the tumor microenvironment, including vascular and immune compartments that can influence tumor progression and dissemination. Tumor vascular remodeling is reinforced by PDLIM5-dependent filopodia formation in endothelial tip cells. PDLIM5 promotes ACTN1/ACTN4-dependent F-actin bundling, facilitating endothelial sprouting and tumor angiogenesis [46].

Beyond classical signaling, tumor–endothelial communication can also involve intercellular mitochondrial transfer. LUAD cells have been shown to transfer mitochondria enriched with specific tsRNAs, such as mt-5′tiRNA-34-GlnTTG, to endothelial cells. The tsRNA binds FUBP3 and facilitates its translocation, enhancing ribosomal assembly efficiency and translation rates and stimulating endothelial proliferation and migration, thereby promoting LUAD metastasis [47].

Hypoxia can also remodel the immune microenvironment through tumor-derived exosomal signaling. In NSCLC, hypoxia-derived tumor exosomes containing NDUFV2 enhance macrophage mitochondrial function, including OXPHOS, ATP production, and mitochondrial membrane potential, while suppressing macrophage ferroptosis and promoting M2 polarization [48]. A complementary systemic example is provided by intermittent hypoxia associated with obstructive sleep apnea, which increases CD25 and soluble CD25 (sCD25) through HIF-1α-dependent mechanisms; elevated sCD25 has also been associated with tumor aggressiveness and mortality risk in patients with lung cancer and sleep apnea [49]. Conversely, modulation of the redox microenvironment can influence macrophage polarization. In NSCLC models, catalase-mediated reduction in oxidative stress promotes a shift of tumor-associated macrophages from an M2 toward an M1 phenotype and is associated with reduced tumor growth [50].

### 2.6. Clinical Implications of Hypoxia-Driven Specialization

The progressive specialization of mitochondrial metabolism during hypoxia has opened the possibility for diagnostic and therapeutic options. In peripheral lung cancer, HIF-1α, GLUT1, and LUNX mRNA expression in brush-cytology samples was detected significantly more frequently in patients with cancer than in those with benign lesions, and positive expression was associated with poorer prognosis, suggesting potential biomarker value [51]. Additionally, the Vascular Risk Score (VRS) provides a noninvasive, imaging-based measure of tumor vascular abnormality and has improved prediction of radiotherapy response in NSCLC compared with vessel density [52].

Emerging imaging technologies, including chemiluminescence-based probes, offer experimental approaches to detect ROS and other redox-active species in the tumor microenvironment, although their application to clinical patient stratification remains investigational [53].

Collectively, these findings support the relevance of hypoxia-associated biomarkers and emerging imaging approaches in lung cancer, while highlighting the potential translational relevance of processes associated with tumor adaptation to hypoxic stress. Despite profound environmental stress, including hypoxia, tumor populations can coordinate adaptations involving metabolic reprogramming, redox control, mitochondrial proteostasis, angiogenesis, and immune modulation. Within the proposed AMS framework, these processes illustrate complementary dimensions of stress adaptation that may reveal metabolic and redox vulnerabilities with potential value as therapeutic targets or biomarkers. However their clinical utility requires prospective validation.

## 3. Mitochondrial Proteome Remodeling and Inter-Organelle Communication in Lung Adenocarcinoma

**Figure 2:**
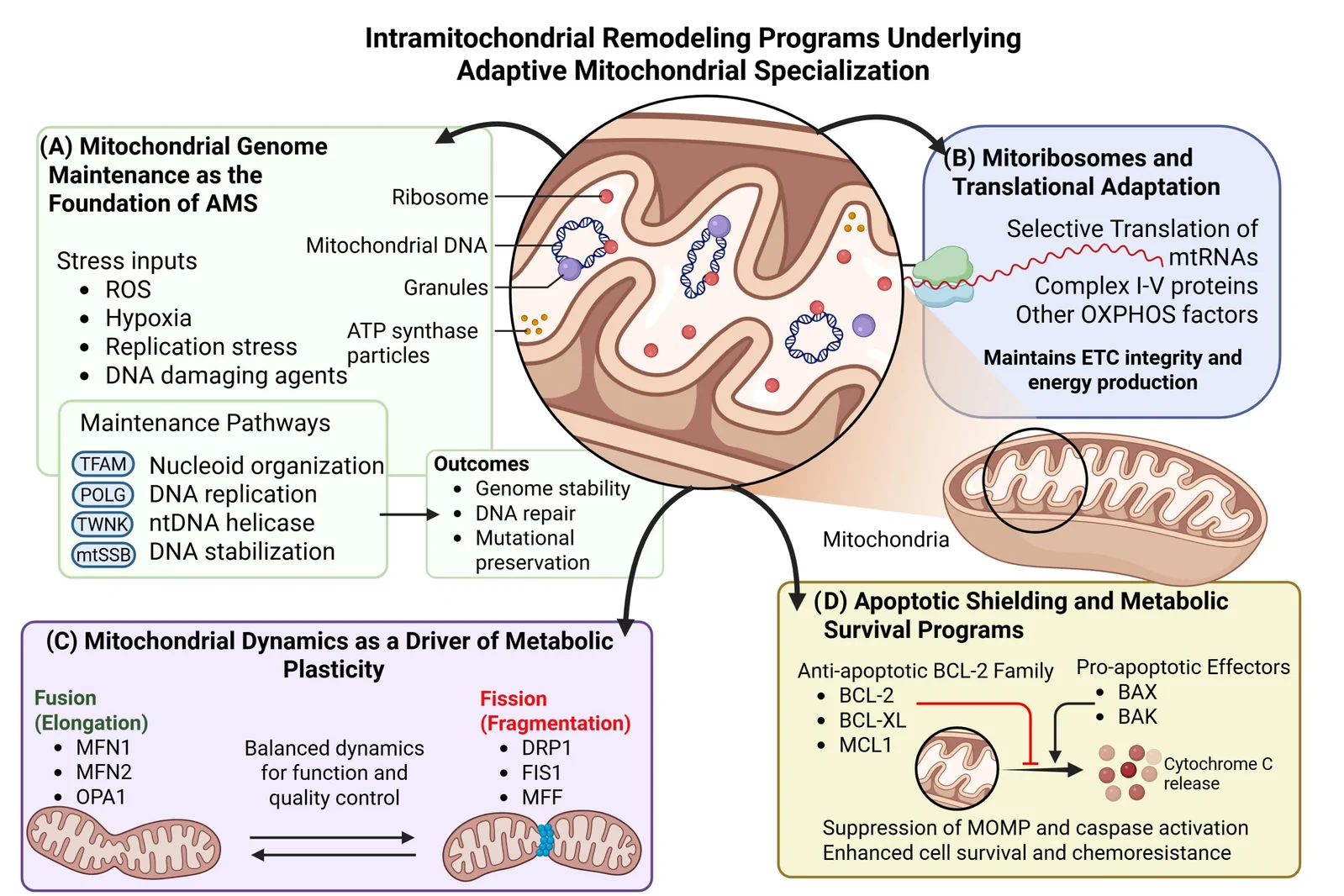
Key Intramitochondrial Mechanisms of AMS. (**A**) The mitochondrial genome can be challenged by various stressors, including reactive oxygen species (ROS), hypoxia, replication stress and other DNA-damaging agents. Mitochondrial genome-maintenance mechanisms involving TFAM, POLG, TWNK, and mtSSB help preserve mtDNA integrity and relate to the genome-maintenance component of AMS. (**B**) Mitoribosomal function and mitochondrial translation support the synthesis of mitochondrially encoded OXPHOS subunits, thereby contributing to respiratory-chain integrity and mitochondrial bioenergetic capacity. (**C**) Various proteins regulate mitochondrial dynamics through context-dependent control of fusion and fission, supporting mitochondrial function, quality control, and metabolic plasticity. (**D**) The balance between anti-apoptotic BCL-2 family proteins and pro-apoptotic effectors regulates mitochondrial apoptotic susceptibility. Anti-apoptotic signaling can limit mitochondrial outer membrane permeabilization (MOMP), cytochrome c release, and subsequent caspase activation, thereby supporting cell survival and chemoresistance. Created in BioRender. Aquino-gálvez, A. (2026) https://BioRender.com/zmryi8u.

Preservation of mitochondrial function during environmental stress requires coordinated maintenance of mitochondrial gene expression, mtDNA homeostasis, and the mitochondrial translational machinery (Figure 2). Within the proposed AMS framework, these processes can support the mitochondrial functional capacity required for subsequent metabolic adaptation. POLRMT, an essential component of mitochondrial gene expression, is overexpressed in LUAD [5]. In contrast, DGUOK maintains mtDNA homeostasis and supports Complex I biogenesis and OXPHOS, thereby contributing to cancer stem-cell self-renewal in LUAD [6]. Mitochondrial functional capacity can be further influenced by PGC-1α, a regulator of mitochondrial biogenesis and oxidative metabolism that is elevated in p53-WT LUAD cells and supports their proliferation [42]. Collectively, these mechanisms support mitochondrial functional capacity and may contribute to tumor-cell adaptation and progression.

### 3.1. Remodeling of the Mitochondrial Proteome in LUAD

Alongside mtDNA maintenance, mitochondrial proteome remodeling can alter bioenergetic, redox, and stress-response functions in LUAD cells.

Among proteins associated with mitochondrial proteome regulation, TIMM17A, a component of the mitochondrial protein-import machinery, is upregulated in LUAD, and its expression correlates with tumor progression and poor prognosis; TIMM17A knockdown also reduces LUAD cell growth [54]. Meanwhile, OCIAD2 promotes glycolytic reliance while suppressing mitochondrial oxidative metabolism, thereby supporting LUAD progression. In the Taiwan Cancer Moonshot cohort, 95.5% of LUAD tumors showed elevated OCIAD2 protein expresion, which was associated with poor prognosis [55].

Recent evidence further demonstrates that post-translational modification of mitochondrial metabolic enzymes can coordinate bioenergetic and redox remodeling in LUAD. Increased K409 succinylation of dihydrolipoamide S-succinyltransferase (DLST), catalyzed by CPT1A, modulates DLST activity and affects OXPHOS and redox homeostasis, thereby promoting LUAD progression and resistance to cuproptosis [56].

SIRT3 provides an additional example of context-dependent mitochondrial proteome regulation. Proteomic and acetylomic analyses after pharmacological SIRT3 inhibition in A549 LUAD cells revealed altered mitochondrial metabolic pathways and increased oxidative metabolism, indicating that the proteomic consequences of SIRT3 modulation depend on the cellular metabolic context [57].

Likewise, BAG5 integrates metabolic and mitochondrial-dynamic adaptations in NSCLC. BAG5 supports glycolytic metabolism through post-transcriptional regulation of GLUT3, whereas BAG5 loss reduces glucose uptake, lactate production, and glycolytic activity while increasing oxidative metabolism. Proteomic analyses suggest potential interactions with RNA-binding proteins, including IGF2BP1–3, although their causal role in GLUT3 regulation remains to be established. BAG5 also influences mitochondrial dynamics, as its loss increases MFN2, decreases DRP1, and promotes a more fused mitochondrial morphology [58].

Collectively, these findings support active remodeling of mitochondrial proteomic, metabolic, and structural programs in LUAD/NSCLC, consistent with the coordinated mitochondrial adaptation proposed within the AMS framework. Key mitochondrial proteins and their functional relevance to AMS are summarized in Table 1.

### 3.2. Mitoribosomes and Translational Adaptation

Dysregulation of mitochondrial ribosomal proteins (MRPs) has been reported in LUAD. As components of the mitochondrial translation machinery, MRPs support synthesis of mtDNA-encoded respiratory-chain subunits, and individual MRPs are also associated with tumor-related processes, including proliferation, apoptosis, cell-cycle regulation, EMT, invasion, and DNA-damage responses [61]. MRPL51 is upregulated in LUAD and is associated with unfavorable clinicopathological features and poor prognosis. Experimental evidence further indicates that MRPL51 promotes LUAD-cell proliferation and invasion, potentially through the NTS/EGFR/PI3K/AKT signaling axis [62,63]. Similarly, MRPL13 expression is associated with cell-cycle regulation, EMT, invasion, and metastatic phenotypes in LUAD [64], whereas MRPL19 is associated with cell-cycle-related pathways, lymph-node metastasis, and poor prognosis [65].

MRPL12 is upregulated in LUAD and is associated with poor survival, while experimental overexpression promotes proliferation, migration, invasion, tumorigenesis, and metastasis. Mechanistically, phosphorylation of MRPL12 at Y60 promotes its interaction with POLRMT and supports mitochondrial metabolic reprogramming toward OXPHOS. Conversely, UBASH3B-mediated dephosphorylation of MRPL12 at Y60 weakens its interaction with POLRMT and suppresses its oncogenic activity [66]. Finally, MRPS24 is overexpressed in LUAD, with its expression inversely associated with both MRPS24 methylation levels and immune-cell infiltration [67]. Collectively, these findings indicate that mitoribosomal remodeling in LUAD extends beyond mitochondrial translation and can intersect with bioenergetic regulation, oncogenic signaling, and the tumor immune microenvironment. Within the proposed AMS framework, these observations support mitochondrial translational regulation as a context-dependent component of mitochondrial adaptation rather than as an isolated marker of AMS.

### 3.3. Mitochondrial Dynamics as a Driver of Metabolic Plasticity

Beyond proteome remodeling, LUAD progression is also associated with alterations in mitochondrial dynamics, architecture, and ROS signaling. In cancer-cell models, prolonged hypoxia can induce HIF-1-dependent, MFN1-mediated mitochondrial fusion and enlargement, contributing to resistance to apoptotic stimuli [68]. Conversely, increased DRP1 activity can favor mitochondrial fragmentation in proliferating LUAD cells, and DRP1 expression and activation have been associated with proliferation and disease progression [69]. Consistent with this, MTP18 promotes excessive mitochondrial fission in LUAD, increasing ROS accumulation and activating PI3K/AKT signaling to support proliferation and metastasis [1]. BAG5 also contributes to mitochondrial fragmentation; loss of BAG5 decreases DRP1, increases MFN2, and promotes a more fused mitochondrial morphology [58]. In NSCLC, absent in melanoma 2 (AIM2) is overexpressed and colocalizes with mitochondria. AIM2 depletion increases MFN2 expression and mitochondrial fusion, reducing ROS production and downstream MAPK/ERK signaling [70].

Furthermore, in lung cancer models, fascin is recruited to mitochondria under metabolic stress, where stabilized mitochondrial F-actin promotes mtDNA homeostasis, Complex I biogenesis, and OXPHOS, thereby supporting metabolic-stress resistance and metastatic colonization [59]. Collectively, these findings indicate that mitochondrial fusion and fission are dynamically regulated in a context-dependent manner and can influence bioenergetics, ROS signaling, proliferation, and metastatic fitness. Within the proposed AMS framework, mitochondrial dynamics therefore represents a structural mechanism that can support metabolic and redox adaptation rather than a uniformly pro-fission or pro-fusion phenotype.

### 3.4. Apoptotic Shielding and Metabolic Survival Programs

Within the proposed AMS framework, adaptive mitochondrial remodeling can include mechanisms that limit the activation of mitochondria-dependent apoptosis under metabolic, oxidative, or therapeutic stress.

Protein tyrosine phosphatase receptor type O (PTPRO), a tumor suppressor, is downregulated in LUAD. Experimental PTPRO overexpression decreases anti-apoptotic Bcl-2, increases Bax, Bid, cleaved caspase-3/9, and cleaved PARP, and promotes mitochondrial dysfunction and mitochondria-dependent apoptosis [71]. In cancer-cell models, mitochondrial HK2 binding through VDAC can reduce susceptibility to apoptosis by interfering with Bax-mediated mitochondrial events, including cytochrome c release [72]. In EGFR-mutant NSCLC cells with acquired gefitinib resistance, researchers have reported increased mitochondrial bioenergetics, a shift from glycolytic dependence toward greater OXPHOS utilization and increased MCT1 expression. Pharmacological inhibition of MCT1 with AZD3965 reduces cell proliferation and motility, identifying metabolic adaptation as a potential vulnerability in TKI-resistant cells [73].

Collectively, these findings illustrate how mitochondrial apoptotic control and metabolic reprogramming can converge to support tumor-cell survival under therapeutic stress. Within the proposed AMS framework, apoptotic shielding therefore represents a context-dependent survival component of mitochondrial adaptation rather than a universal consequence of metabolic remodeling.

### 3.5. Inter-Organelle Communication and the mTORC2-MAM Axis

Inter-organelle communication between mitochondria and the endoplasmic reticulum (ER) adds another layer of metabolic and stress adaptation relevant to LUAD. This interaction takes place at the specialized contact sites known as mitochondria-associated membranes (MAMs) that participate in bioenergetic regulation, lipid metabolism, calcium signaling, mitochondrial metabolism, redox homeostasis, and apoptosis by functionally coupling the ER and mitochondria, MAMs provide an interface through which Ca^2+^ transfer, lipid metabolism, mitochondrial bioenergetics, redox signaling, and cell-death susceptibility can be coordinated in response to cellular stress [74].

Recently, through transcriptomic analysis integrating data from the Cancer Genome Atlas (TCGA), Zhang et al. developed a prognostic model based on a MAM-centered gene signature, identifying that elevated expression of the MAM-associated genes ERO1A, SHC1, and CCT6A correlated with notably; through pathway enrichment analysis, these MAM-related genes were associated with immune regulatory pathways and patterns of immune-cell infiltration, including CD8+ and CD4+ T cells, B cells, and macrophages [74]. These findings support the clinical relevance of MAM-associated programs in LUAD. However, these transcriptomic associations do not establish a causal role for MAM remodeling or mTOR signaling in tumor progression.

Establishing a mechanistic link between MAM signaling and the mTOR network requires distinguishing mechanistic evidence from LUAD-specific observations. Mechanistically, studies outside the LUAD-specific setting have demonstrated that mTOR complex 2 (mTORC2) localizes to MAMs in a growth factor-dependent manner and interacts with the IP3R–GRP75–VDAC1 ER–mitochondrial tethering complex. mTORC2 deficiency disrupts MAM integrity and alters mitochondrial physiology, increasing mitochondrial Ca^2+^ uptake, membrane potential, and ATP production, supporting a role for mTORC2–Akt signaling in regulating ER–mitochondrial communication and mitochondrial physiology [75]. Although direct evidence establishing an equivalent mTORC2–MAM regulatory axis in LUAD remains limited, these mechanistic observations provide a biologically relevant framework for interpreting how MAM-associated signaling may integrate Ca^2+^ homeostasis, bioenergetics, and survival pathways in tumor cells. Within the AMS framework, the mTORC2–MAM axis should therefore be considered a candidate mechanism of inter-organelle integration rather than a universal or LUAD-specific defining feature of AMS.

The role of calcium signaling in ER–mitochondrial communication in NSCLC is further supported by studies of the sarco/endoplasmic reticulum Ca^2+^-ATPase 2 (SERCA2). SERCA2 is a major regulator of ER Ca^2+^ homeostasis, and pharmacological inhibition with diphyllin in NSCLC cells has been associated with reduced proliferation and migration, along with increased apoptosis. SERCA2 inhibition impairs Ca^2+^ reuptake into the ER, increasing cytosolic Ca^2+^ and subsequent mitochondrial Ca^2+^ uptake, promoting mitochondrial dysfunction characterized by increased ROS production, loss of mitochondrial membrane potential, and cytochrome c release [76]. Together, these observations support ER–mitochondrial communication as a functionally relevant component of tumor metabolic adaptation. MAMs provide an interface through which Ca^2+^ handling, mitochondrial bioenergetics, redox signaling, and apoptotic susceptibility can be coordinated, while mTORC2–Akt signaling represents one mechanism capable of modulating this interface. Within the AMS framework, MAM remodeling is therefore relevant not as an isolated molecular alteration, but as part of the coordinated inter-organelle integration through which mitochondrial function can be adjusted to environmental and metabolic stress.

### 3.6. P Mitochondria-to-Nucleus Communication and Epigenetic Remodeling

**Figure 3:**
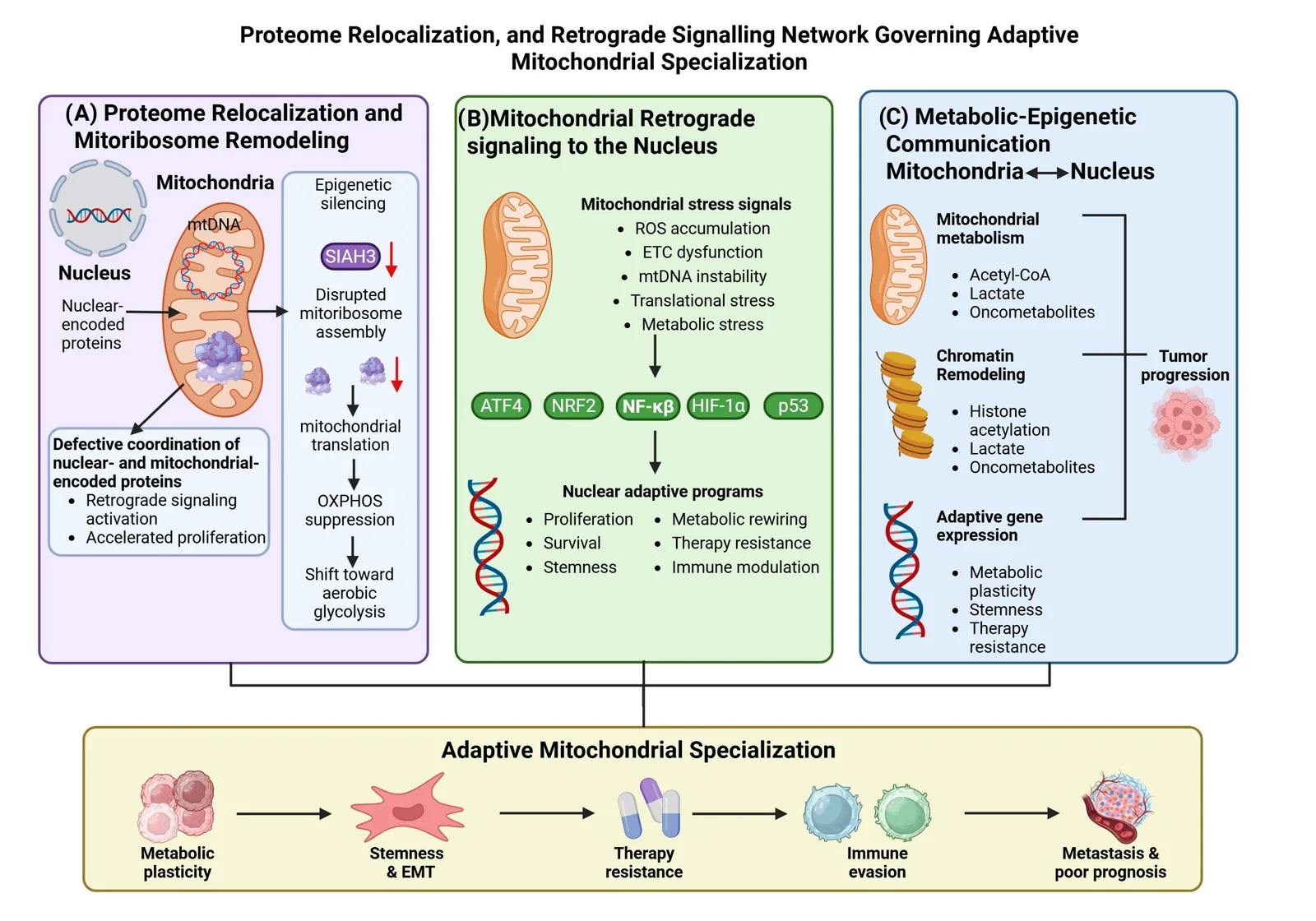
Mitochondrial–nuclear communication associated with AMS. (**A**) Seven in absentia homolog 3 (SIAH3) is epigenetically silenced in several cancers, including LUAD, and localizes to mitochondria, where it interacts with proteins involved in mitoribosome biogenesis and mitochondrial translation. Experimental SIAH3 expression suppresses mitochondrial respiration and shifts cellular metabolism from oxidative phosphorylation (OXPHOS) toward glycolysis, illustrating an association between a mitochondria-localized, epigenetically regulated protein and cellular metabolic state [77]. (**B**) Mitochondrial stressors can engage transcriptional regulators and nuclear adaptive programs that support survival, stemness, proliferation, and other cellular processes. (**C**) Mitochondrial metabolism can influence epigenetic regulation by providing metabolites that modulate histone acetylation, DNA and histone methylation, and chromatin remodeling, thereby providing a mechanism through which metabolic state can influence nuclear gene regulation. Together, these mechanisms illustrate complementary routes through which mitochondrial remodeling may communicate with nuclear programs and contribute to metabolic plasticity and broader tumor-adaptive responses. Within the AMS framework, these processes are interpreted as context-dependent components of mitochondrial adaptation rather than as universal features that must occur simultaneously in every LUAD cell. Created in BioRender. Aquino-gálvez, A. (2026) https://BioRender.com/5telxyt.

Mitochondrial adaptation is bidirectionally connected to nuclear regulation through changes in mitochondrial protein expression, metabolic state, and metabolite availability (Figure 3). In LUAD, SIAH3 is frequently epigenetically silenced in LUAD and other cancer types and localizes to mitochondria, where it interacts with proteins involved in mitochondrial ribosome biogenesis and translation. Importantly, inducing SIAH3 expression shifts cellular metabolism from oxidative phosphorylation to glycolysis, reduces cell proliferation, and promotes cell death [77]. These findings illustrate how epigenetic regulation of a mitochondrial protein can reshape tumor-cell bioenergetics. Conversely, mitochondrial metabolism can influence nuclear gene regulation by controlling the availability of metabolites that serve as substrates or cofactors for chromatin-modifying enzymes. Acetyl-CoA availability provides a direct metabolic link between cellular metabolism and histone acetylation [78]. Consistent with this metabolic–epigenetic connection, mitochondrial function has been shown to maintain histone acetylation, particularly H3K27ac, in colorectal cancer cells [79]. Although these mechanisms have not been established as a unified pathway in LUAD, they provide mechanistic evidence that changes in mitochondrial metabolic state can influence nuclear chromatin regulation.

A related mechanism involves TCA-cycle-derived metabolites and oncometabolites. Succinate and fumarate can competitively inhibit α-ketoglutarate-dependent dioxygenases, including histone demethylases and TET-family enzymes, thereby altering histone and DNA methylation [80]. Similarly, the oncometabolite 2-hydroxyglutarate (2-HG) can inhibit α-ketoglutarate-dependent dioxygenases, thereby altering epigenetic regulation and cellular differentiation [81]. Because these mechanisms were established in tumor contexts outside LUAD, they should be interpreted as mechanistic support for metabolic–epigenetic communication rather than as direct evidence of an LUAD-specific AMS pathway.

Within the AMS framework, mitochondria-to-nucleus communication should therefore be considered a context-dependent component of adaptive integration rather than a single LUAD-specific pathway. Changes in mitochondrial metabolism, translation, and metabolite availability can influence nuclear and epigenetic programs, while nuclear and epigenetic regulation can reciprocally reshape mitochondrial function. This bidirectional communication provides a mechanistic basis for integrating mitochondrial adaptation with broader transcriptional and cellular stress responses.

## 4. Therapeutic Opportunities Emerging from Adaptive Mitochondrial Specialization

The coordinated mitochondrial adaptations encompassed by the AMS framework may also expose context-dependent therapeutic vulnerabilities. Rather than indiscriminately targeting mitochondrial function, these strategies aim to exploit specific bioenergetic, redox, or stress-response dependencies acquired during tumor adaptation.

This reprogramming is driven by both environmental risk factors and infectious challenges and organelle-to-organelle signaling. For example, exposure of lung adenocarcinoma cells to the tobacco-specific nitrosamine NNK stimulates mitochondrial function and biogenesis through β2-adrenergic receptor-associated signaling, illustrating how an environmental carcinogen can promote mitochondrial metabolic remodeling [82]. Mechanistic studies outside the tumor-cell setting further illustrate how inter-organelle stress can remodel mitochondrial function. In macrophages, lysosomal damage induces cathepsin-dependent proteolytic remodeling of the mitochondrial proteome and metabolic reprogramming independently of mitophagy; in vivo, lysosomal damage is also associated with altered mitochondrial function and metabolic and immune responses in lung macrophages [83]. Under glucose deprivation, PFKP acquires a noncanonical adaptive role by facilitating mitochondrial recruitment of AMPK, which promotes ACC2 phosphorylation and long-chain fatty-acid oxidation, thereby supporting energy and redox homeostasis and NSCLC-cell survival [84].

Adaptive responses can also contribute to resistance to targeted therapies. In H1975 NSCLC cells with acquired resistance to the EGFR inhibitor AZD9291 (osimertinib), kinome profiling using a TK activity-representing peptide library coupled to multiple-reaction monitoring (TARPL-MRM) identified SRC activity as a contributor to the resistant phenotype, and SRC inhibition showed potential to overcome acquired TKI resistance [85].

In response to this adaptive machinery, precision bioenergetic medicine strategies are emerging. In human LUAD, bioenergetic stratification has identified tumors with high mitochondrial respiration (OX+) and increased expression of the mitochondrial trifunctional protein (MTP/HADHA). Genetic inhibition of MTP or pharmacological targeting with trimetazidine impaired OX+ tumor growth by disrupting the MTP–Complex I interaction and inducing a redox and energy crisis [86]. Similarly, pharmacological suppression of mitochondrial superoxide production with the S1QEL compound AOL produces antitumor effects and extensive proteomic remodeling in human lung adenocarcinoma models, including increased expression of the Complex I subunit NDUFV1 [87]. More recently, STING has been shown to stabilize the mitochondrial desuccinylase SIRT5, promoting MDH2 desuccinylation at K314. This modification impairs MDH2 activity and mitochondrial respiration, promotes mtDNA damage and cGAS–STING signaling, and restores cisplatin sensitivity in resistant NSCLC models [88]. Collectively, these studies indicate that mitochondrial adaptation can generate therapeutically exploitable dependencies involving substrate utilization, respiratory activity, redox control, and mitochondrial post-translational regulation. Within the AMS framework, these vulnerabilities should be interpreted as context-dependent therapeutic opportunities rather than as universal targets shared by all LUAD tumors. Therapeutic strategies and targets directed against adaptive mitochondrial specialization and redox vulnerabilities in LUAD are summarized in Table 2.

### 4.1. Mitochondrial Quality Control and the Systemic Consequences of AMS

Although AMS is operationally defined at the tumor-cell level, mitochondrial remodeling during cancer progression may have consequences that extend beyond malignant cells to distant metabolic tissues and tumor-infiltrating immune cells. Mitochondrial quality control is a key component of mitochondrial adaptation to metabolic and oxidative stress. This network integrates mitophagy, mitochondrial biogenesis, fusion/fission dynamics, mitochondrial gene expression regulation to preserve organelle integrity and functional capacity. In respiratory diseases and lung cancer, alterations in these processes have been associated with oxidative stress, apoptosis, cellular senescence, and disease progression. Key regulators include PINK1, Parkin (PRKN), DRP1, and PGC-1α, supporting mitochondrial quality control as an integrated regulatory network rather than a single pathway [20].

Recent LUAD-specific evidence further supports this network´s relevance to tumor-cell adaptation. UBR4, an E3 ubiquitin ligase in the N-degron pathway, has been implicated in mitophagy-associated mitochondrial homeostasis in LUAD, and UBR4 loss promotes senescence associated with defective mitochondria while suppressing tumor growth in experimental models [92]. Beyond these tumor-cell-intrinsic mechanisms, mitochondrial quality-control alterations may also accompany systemic and immune remodeling during cancer progression.

In Lewis lung carcinoma (LLC)-bearing mice, hepatic mitochondrial quality-control regulators changed before detectable changes in collagen deposition, and tumor progression was later associated with increased collagen deposition and hepatic fibrosis [93]. These findings suggest that mitochondrial quality-control alterations may precede tissue pathology during cancer cachexia, although they do not establish a direct causal relationship between mitochondrial dysfunction and fibrosis.

Skeletal muscle provides further evidence for this temporal relationship. In male LLC-bearing mice, mitochondrial degeneration preceded measurable muscle wasting and was accompanied by alterations in regulators of mitochondrial biogenesis, fusion/fission, and mitophagy [94]. Complementary studies in female LLC-bearing mice identified mitochondrial degeneration, increased BNIP3, reduced PGC-1α and TFAM, and impaired muscle contractile function during cachexia [95]. Together, these findings indicate that mitochondrial quality-control remodeling accompanies skeletal-muscle dysfunction and wasting, and in male LLC-bearing mice, can precede measurable muscle atrophy. The distinct temporal pattern observed in female tumor-bearing mice further suggests sex-dependent differences in the development of cachexia-associated mitochondrial alterations. Mitochondrial quality control is also relevant to antitumor immunity. In exhausted CD8+ T cells, prolonged antigen stimulation activates TCR–NFATc1 signaling and upregulates the mitochondrial deubiquitinase USP30, which inhibits mitophagy. Genetic deletion or pharmacological inhibition of USP30 restores mitophagy, improves mitochondrial fitness and effector function, and enhances antitumor responses in experimental models [91]. Because these observations were not established specifically in LUAD, USP30 should not be considered a LUAD-specific determinant of AMS. Rather, they demonstrate how impaired mitochondrial quality control can compromise the metabolic fitness of tumor-infiltrating T cells.

Tumor cells may further influence antitumor immunity through intercellular mitochondrial transfer. Tumor-associated mtDNA mutations have been identified in tumor-infiltrating lymphocytes, and experimental studies show that cancer cells transfer mitochondria carrying tumor-derived mtDNA mutations to T cells. These transferred mitochondria resist mitophagy, and their acquisition is associated with metabolic abnormalities, cellular senescence, impaired effector function and memory formation, and reduced antitumor immunity [96]. Clinical analyses further linked tumor mtDNA mutations to poorer outcomes after immune-checkpoint inhibition in melanoma and NSCLC. This mechanism has not been established specifically in LUAD and should therefore be interpreted as evidence of a tumor–immune mitochondrial interaction rather than a defining feature of AMS.

Importantly, these systemic and immune manifestations should remain conceptually distinct from the operational definition of AMS. AMS refers specifically to the coordinated adaptive specialization of mitochondrial function within tumor cells, integrating bioenergetic reprogramming, redox control, mitochondrial architecture and quality control, and context-dependent inter-organelle communication. Mitochondrial remodeling in liver, skeletal muscle, and immune cells, as well as intercellular mitochondrial transfer, should therefore be regarded as downstream consequences, interacting mechanisms, or tumor–host manifestations associated with cancer-related mitochondrial remodeling rather than independent diagnostic criteria for AMS.

### 4.2. Therapeutic Limitations

Despite promising preclinical evidence, translating mitochondrial and metabolic vulnerabilities into effective therapies remains challenging. Telaglenastat failed to demonstrate clinical benefit in the phase II KEAPSAKE trial (NCT04265534). In contrast, the Complex I inhibitor IACS-010759 showed a narrow therapeutic window, with dose-limiting metabolic and neurological toxicities in phase I trials [97]. Other strategies discussed here, including MTP/HADHA, GPX4, and USP30 targeting, remain predominantly preclinical and therefore require further validation. These limitations do not negate the biological adaptations encompassed by AMS, but emphasize that a mitochondrial dependency is not necessarily a clinically actionable vulnerability. Metabolic plasticity, tumor heterogeneity, and toxicity to normal tissues may further limit therapeutic efficacy. Accordingly, translation of AMS-associated vulnerabilities will require appropriate patient stratification and selective targeting of context-specific dependencies.

## 5. Conclusions and Perspectives

Adaptive mitochondrial specialization (AMS) offers an integrative framework for understanding how mitochondrial function can be selectively remodeled, rather than uniformly suppressed, in LUAD under hypoxia, nutrient limitation, therapeutic pressure, and other microenvironmental stressors.By coordinating bioenergetic metabolism, redox homeostasis, mitochondrial architecture and dynamics, proteostasis and mitochondrial quality control, mtDNA maintenance and mitochondrial translation, apoptotic susceptibility, and inter-organelle communication, tumor cells can preserve mitochondrial functions that support adaptation and survival under stress. These responses may also contribute, in a context-dependent manner, to tumor progression and therapeutic resistance.

Within the AMS framework, metabolic adaptation may include hybrid states in which increased glycolytic activity coexists with residual or functionally sufficient mitochondrial metabolism, including OXPHOS, substrate oxidation, mitochondrial gene expression, biosynthetic functions, and redox control. Importantly, such hybrid metabolic states are not by themselves sufficient to define AMS; rather, AMS requires coordinated adaptation across multiple mitochondrial functional domains. This distinction separates AMS from metabolic plasticity or hybrid glycolytic/OXPHOS phenotypes considered in isolation. This framework therefore highlights mitochondrial bioenergetic, redox, proteostatic, structural, quality-control, apoptotic, and inter-organelle dependencies as potential context-specific therapeutic vulnerabilities. However, AMS should not be interpreted as a single molecular pathway, biomarker, or universally conserved mitochondrial phenotype. Its experimental validation will require an integrated assessment of multiple mitochondrial domains under defined genetic, environmental, and therapeutic contexts. Future studies should determine whether such coordinated adaptive states can be prospectively identified in human LUAD, and whether disrupting critical nodes within these integrated networks can improve therapeutic responses.

## Figures and Tables

**Table 1:** Representative mitochondrial and metabolic adaptations relevant to the AMS framework in lung adenocarcinoma.

Protein/Axis	Experimental Context	Main Reported Finding	Functional Relevance to AMS	Ref.
POLRMT	LUAD	↑ POLRMT; associated with WNT/β-catenin signaling	Mitochondrial gene- expression capacity	[5]
DGUOK	LUAD	DGUOK → mtDNA homeostasis, Complex I biogenesis, OXPHOS	Mitochondrial genome/function maintenance	[6]
TIMM17A	LUAD	↑ TIMM17A; associated with tumor progression	Mitochondrial protein-import machinery	[54]
OCIAD2	LUAD	↑ OCIAD2 → ↓ OXPHOS; ↑ glycolytic reliance	Bioenergetic reprogramming	[55]
CPT1A–DLST	LUAD	↑ DLST K409 succinylation → modulation of OXPHOS and redox homeostasis; ↑ cuproptosis resistance	Post-translational metabolic/redox remodeling	[56]
Fascin	Lung cancer models	Fascin → ↑ mtF-actin, mtDNA homeostasis, Complex I biogenesis, OXPHOS	Structural/bioenergetic remodeling	[59]
CPS1	LKB1-inactivated LUAD	↑ CPS1; supports tumor-cell growth and nucleic-acid biosynthesis-associated metabolism	Genotype-dependent metabolic adaptation	[8]
PDIA4/PDIA6	Cisplatin- resistant NSCLC/LUAD	↑ PDIA4/PDIA6 in cisplatin-resistant cells; their inhibition enhances cisplatin-induced cell death	ER proteostasis–cell-death coupling	[60]
PLCγ1–KRAS	KRAS-mutant LUAD, hypoxia	↓ PLCγ1 → ↓ mitochondrial Ca^2+^/mtROS and lipid peroxidation; ↑ glycolysis	Ca^2+^–redox–metabolic integration	[9]
PGC1-α	p53-WT LUAD	↑ PGC-1α; supports proliferation	Mitochondrial metabolic regulation/bioenergetics	[42]
SIRT3	A549 LUAD cells	SIRT3 inhibition alters mitochondrial metabolic pathways and increases oxidative metabolism	Context-dependent mitochondrial metabolic/proteomic regulation	[57]
BAG5	NSCLC	BAG5 supports GLUT3-associated glycolytic metabolism; BAG5 loss ↑ MFN2, ↓ DRP1 and promotes mitochondrial fusion	Metabolic–mitochondrial dynamics integration	[58]

Representative mitochondrial and metabolic adaptations relevant to the AMS framework in lung adenocarcinoma. ↑, increased expression, abundance, or activity; ↓, decreased expression, abundance, or activity; →, promotes, supports, or shifts toward the indicated process, as reported in the cited experimental context. Abbreviations: AMS, adaptive mitochondrial specialization; LUAD, lung adenocarcinoma; mtDNA, mitochondrial DNA; mtROS, mitochondrial reactive oxygen species; NSCLC, non-small-cell lung cancer; OXPHOS, oxidative phosphorylation; WT, wild type. The listed alterations represent context-dependent mechanisms relevant to individual AMS domains and should not be interpreted as universal features or as individually sufficient evidence of an AMS state.

**Table 2:** Targeting adaptive mitochondrial specialization in LUAD.

Therapeutic Target	Drug/Agent	Status	AMS-Associated Vulnerability	Experimental/ Clinical Context	Ref.
GLS	Telaglenastat (CB-839) + chemoimmunotherapy	Phase II; terminated for lack of clinical benefit	Glutamine dependence	Metastatic KEAP1/NRF2-mutant nonsquamous NSCLC	NCT04265534 [89]
MTP/HADHA–FAO	Trimetazidine	Preclinical/repurposing	Energy/redox dependence	OX+ human lung adenocarcinoma	[86]
GPX4	RSL3	Preclinical	Lipid-peroxidation/ferroptosis vulnerability	EGFR-TKI-resistant LUAD	[19]
Complex I/OXPHOS	IACS-010759 +trametinib	Preclinical combination; IACS-010759 evaluated separately in phase I trials	MEK-inhibitor-induced OXPHOS dependence	KRAS-mutant NSCLC	[90]
USP30/mitophagy	USP30 inhibition	Preclinical	Impaired mitochondrial quality control/mitophagy	Exhausted tumor-infiltrating CD8+ T cells; non-LUAD-specific	[91]

Therapeutic strategies targeting mitochondrial and metabolic vulnerabilities relevant to the adaptive mitochondrial specialization (AMS) framework. Development status refers to the highest stage of investigation reported for the indicated strategy and does not imply clinical efficacy in LUAD. Preclinical findings from NSCLC, other tumor models, or immune-cell contexts are noted accordingly. AMS, adaptive mitochondrial specialization; FAO, fatty acid oxidation; GLS, glutaminase; GPX4, glutathione peroxidase 4; LUAD, lung adenocarcinoma; MTP, mitochondrial trifunctional protein; NSCLC, non-small-cell lung cancer; OXPHOS, oxidative phosphorylation; USP30, ubiquitin-specific peptidase 30. These interventions should be regarded as strategies targeting vulnerabilities associated with mitochondrial adaptation rather than as validated AMS-specific therapies.

## Data Availability

No new data were created or analyzed in this study. Data sharing is not applicable to this article.

## Abbreviations

Abbreviations: 2-HG, 2-hydroxyglutarate; AKT, protein kinase B; AMPK, AMP-activated protein kinase; AMS, adaptive mitochondrial specialization; ATP, adeno-sine triphosphate; BAG5, BCL2-associated athanogene 5; BNIP3, BCL2 interacting protein 3; CA9, carbonic anhydrase 9; CPS1, carbamoyl-phosphate synthase 1; CSC, cancer stem cell; DGUOK, deoxyguanosine kinase; DRP1, dynamin-related protein 1; EGFR, epidermal growth factor receptor; EMT, epithelial–mesenchymal transition; ER, endoplasmic reticulum; FAO, fatty acid oxidation; Fe–S, iron–sulfur; GLS, glutaminase; GLUT1/3, glucose transporter 1/3; GPX4, glutathione peroxidase 4; GSH, reduced glutathione; GSSG, oxidized glutathione; HIF, hypoxia-inducible factor; HK2, hexokinase 2; IDH2, isocitrate de-hydrogenase 2; KRAS, Kirsten rat sarcoma viral oncogene homolog; LLC, Lewis lung carcinoma; LUAD, lung adenocarcinoma; MAM, mitochondria-associated membrane; MCT1, monocarboxylate transporter 1; MFN1/2, mitofusin 1/2; MOMP, mitochondrial outer membrane permeabilization; MRP, mitochondrial ribosomal protein; mtDNA, mitochondrial DNA; MTP, mitochondrial trifunctional protein; mtROS, mitochondrial reactive oxygen species; mTORC1, mechanistic target of rapamycin complex 1; mTORC2, mechanistic target of rapamycin complex 2; NADPH, reduced nicotinamide adenine dinucleotide phosphate; NNT, nicotinamide nucleotide transhydrogenase; NSCLC, non-small-cell lung cancer; OXPHOS, oxidative phosphorylation; PGC-1α, peroxisome proliferator-activated receptor gamma coactivator 1-alpha; PI3K, phosphoinositide 3-kinase; POLRMT, mitochondrial RNA polymerase; ROS, reactive oxygen species; SERCA2, sarco/endoplasmic reticulum Ca^2+^-ATPase 2; SIRT3, sirtuin 3; SIRT5, sirtuin 5; SOD2, superoxide dismutase 2; STK11/LKB1, serine/threonine kinase 11/liver kinase B1; TCA, tricarboxylic acid cycle; TFAM, mitochondrial transcription factor A; TIMM17A, translocase of inner mitochondrial membrane 17A; TKI, tyrosine kinase inhibitor; TME, tumor microenvironment; TRAP1, TNF receptor-associated protein 1; USP30, ubiquitin-specific peptidase 30; VDAC1, voltage-dependent anion channel 1; YME1L, YME1-like ATPase.
